# High-Quality Genome Assembly and Annotation of the Big-Eye Mandarin Fish (*Siniperca knerii*)

**DOI:** 10.1534/g3.119.400930

**Published:** 2020-01-17

**Authors:** Liang Lu, Jinliang Zhao, Chenhong Li

**Affiliations:** * Shanghai Collaborative Innovation for Aquatic Animal Genetics and Breeding,; † Shanghai Collaborative Innovation for Aquatic Animal Genetics and Breeding, Shanghai Ocean University, Shanghai 201306, China, and; ‡ Key Laboratory of Exploration and Utilization of Aquatic Genetic Resources, (Shanghai Ocean University), Ministry of Education, Shanghai 201306, China

**Keywords:** *Siniperca knerii*, Chinese perch, Genome sequencing, Genome assembly, 10x Genomics

## Abstract

The big-eye mandarin fish (*Siniperca knerii*) is an endemic species of southern China. It belongs to the family Sinipercidae, which is closely related to the well-known North American sunfish family Centrarchidae. Determining the genome sequence of *S. knerii* would provide a foundation for better examining its genetic diversity and population history. A novel sequenced genome of the Sinipercidae also would help in comparative study of the Centrarchidae using Siniperca as a reference. Here, we determined the genome sequence of S. knerii using 10x Genomics technology and next-generation sequencing. Paired-end sequencing on a half lane of HiSeq X platform generated 56 Gbp of raw data. Read assembly using Supernova assembler resulted in two haplotype genomes with 732.1 Mb in size and an average GC content of 40.4%, which is consistent with genome size previously reported or estimated using k-mer counting. A total of 7,989 scaffolds with an N50 score of 12.64 Mb were obtained. The longest scaffold was 30.54 Mb. Evaluation of the genome completeness using BUSCO confirmed that 96.5% genes of the Actinopterygii Benchmarking Universal Single-Copy Orthologs were found in the assembled genome of *S. knerii*. Gene prediction using Maker annotation kit resulted in 28,440 genes, of which 25,899 genes had at least one hit comparing to the NCBI Nr database, KEGG or InterProScan5. Pairwise sequentially Markovian coalescent (PSMC) analysis of the genome showed that there was a bottleneck event of the population of *S. knerii* between 70 ka – 20 ka, which was concordant with the Tali glacier period, suggesting a population decline of S. knerii probably due to climate conditions.

The big-eye mandarin fish (*Siniperca knerii*) is an endemic species of southern China, distributed in drainages such as the Pearl River, the Ming River, the Qiantang River, the Yangtze River and the Huai River (Caiwu *et al.* 1988). *Siniperca knerii* belongs to the family Sinipercidae, which is most closely related to the well-known North American sunfish Centrarchidae (Song *et al.* 2017; Near *et al.* 2012). *Siniperca knerii* is a top piscivorous ambush predator that plays an important role in the ecological health of streams and rivers of southern China (Qiqun *et al.* 2019). The wild population of *S. knerii* is declining in recent years most likely due to overfishing, pollution and hydrological engineering (PU *et al.* 2013). *Siniperca knerii* is also an economically important species to fisheries and aquaculture in southern China (Luo *et al.* 2011). A few studies have employed microsatellite markers, mitochondrial genomes or exon-capture data to study population genetics or to infer the phylogenetic position of *S. knerii* (Chen *et al.* 2014; Song *et al.* 2017; Tian *et al.* 2017), but no genome sequence is available for it or for any species of the Sinipercidae. Determining the genome sequence of *S. knerii* would provide a fundamental dataset for better examining its genetic variation and population history. Whole genome association could also reveal genetic adaptation or assist with establishing selective breeding programs for *S. knerii* for aquaculture purposes. A sequenced genome of the Sinipercidae could also contribute to comparative studies of the Centrarchidae using Siniperca as a reference.

## Materials and Methods

### Sample collection and extraction of high molecular weight genomic DNA (HMW-gDNA)

**Figure 1:**
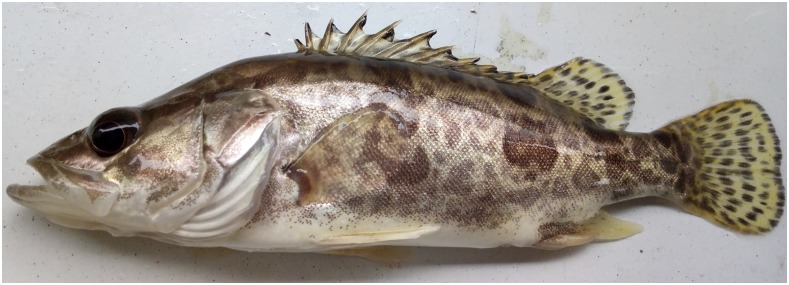
The big-eye mandarin fish (*Siniperca knerii*) collected from Yangzhong, Jiangsu Province of China.

A specimen of *S. knerii* was collected from Yangzhong, Jiangsu Province of China (32°17’40.31”N, 119°50’9.83”E) on Oct. 20, 2017. The specimen was 15 cm in total length (Figure 1). The muscle tissue samples were taken from the specimen and frozen at -80° until further processed in the lab. DNA extraction was carried out within 48 hr using a modified CTAB protocol (Dellaporta *et al.* 1983). The extracted DNA was further purified for large fragments using magnetic beads and inspected with pulsed-field gel electrophoresis https://support.10xgenomics.com/genome-exome/sample-prep/doc/demonstrated-protocol-hmw-dna-extraction-from-fresh-frozen-tissue. Sample with DNA size greater than 50 kb was used for library preparation.

### 10x Genomics library prep, sequencing and K-MER analysis

**Figure 2:**
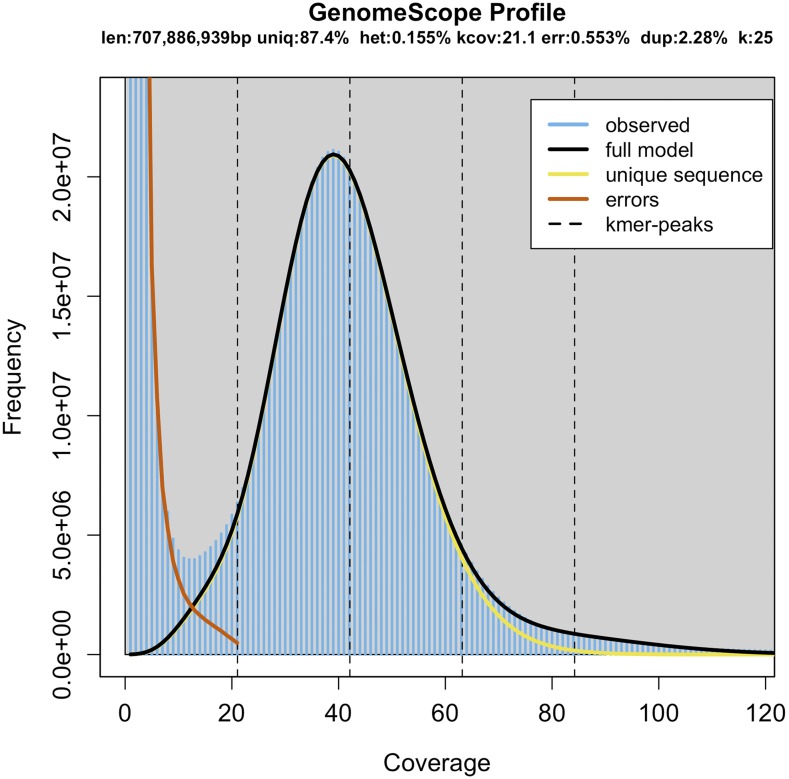
Histogram of the 25-mer depth distribution of the sequencing reads of Siniperca knerii plotted in GenomeScope. The k-mer with a coverage of 40x has the largest number (excluding the k-mer with too low coverage), which was used to calculate the genome size.

Microfluidic partitioned DNA library was made using 10x Genomics system with 0.18 ng of input gDNA. Sequencing was performed as PE150 on a half lane of an Illumina Hiseq X Ten run (Illumina, San Diego, CA, USA). A custom script, cut_10XBarcode.py (File S1) was used to trim the 16bp 10x Genomics barcode. Then, Trimmomatic v0.36 (Bolger *et al.* 2014) was use to exclude low quality reads after trimming. The trimmed reads were only use for k-mer analysis. Frequencies of 25 k-mers were counted using GCE v1.0.0 (BGI, Shenzhen) (Liu *et al.* 2013). GenomeScope v1.0.0 (Vurture *et al.* 2017) was used to estimate size, repeat content and heterozygosity of the genome with maximum k-mer coverage of 10,000. The genome size was calculated as: size = K-mer number/peak depth (Figure 2).

### De novo genome assembly

The paired-end reads were input to the Supernova assembler v2.1.1 (10x Genomics, San Francisco, CA, USA) (Zheng *et al.* 2016) for *de novo* genome assembly. No trimming was needed according to the Supernova assembler original document. The assembly was started under the “supernova run” module. The parameter of maximum reads (-maxreads) was set as 80 based on initial K-MER analysis and empirical adjustment. For better performance of Supernova run on our clusters, the parameter “–localcores” was set as 50, and “–localmem” was set as 1024 GB. Other parameters were left as default. Benchmarking Universal Single-copy Orthologs (BUSCO) v3.0.1 (BUSCO, RRID: SCR_015008) was used for genome completeness assessment (Waterhouse *et al.* 2018). The ”actinopterigii_obd9” dataset containing 20 fish species was used for evaluation (options -m genome -sp zebrafish) and 4584 core genes of vertebrate animals were evaluated.

### Gene prediction and annotation

The assembled genome of *S. knerii* was searched for repetitive sequence using RepeatModeler v1.0.8 (Arian and Robert 2008) with default parameters. The database of repeat sequences constructed using RepeatModeler contains all repeated sequences, and may also contain protein-coding sequences, so the repeat sequences were compared to the perciform genes using blast. The repetitive sequences having hits to protein-coding sequences were excluded from the repeat sequence database for further analyses (File S2). Finally, the genome was subjected to repeated sequence screening using RepeatMasker v4.0.7 (Repeat Masker, RRID: SCR_012954) according to the constructed repetitive sequence database (Arian and Robert 2013–2015). The Maker v2.31.10 (MAKER, RRID: SCR_005309) pipeline was used for gene prediction (Carson and Mark 2011). Models used for gene prediction include *de novo* gene prediction and homology-based gene prediction. Augustus v3.3 (Augustus, RRID: SCR_008417) (Mario and Stephan 2003) implemented in Maker was used to predict *de novo* genes in the genome trained with *Danio rerio* (–species = zebrafish). All genes of the perciforms were retrieved using ”Taxonomy” option from the NCBI database, which were used as references for gene prediction. The *S. chuatsi* full-length transcriptome data from NCBI (BioProject number: PRJNA552987) also were used as references for annotation. Blastn v2.7.1 (BLASTN, RRID: SCR_001598) was used to map the transcriptome to the genome (est2genome parameter in Maker). To get more informative alignments, Exonerate v2. 2 (Slater and Birney 2005) was used to polish blast hits. The resulting gene models were used to train hidden Markov models of Augustus and SNAP Korf (2004)for iterative genome annotation. The iteration was repeated for two more times. Finally, MAKER was used to determine alternative splice forms where EST data permitted, produced quality control metrics for each gene model (included in the output), and then MAKER chose from among all the gene model for the one that best matches the evidence. The gene prediction was compared to well-annotated databases, including InterProScan5 (Philip *et al.* 2014), NCBI Nr, KEGG. The 28,440 predicted genes were split into 24 files for InterProScan5 runs to improve efficiency. A custom script (File S3) was applied to set six processes simultaneously (-p 6) for the runs. The filtered library was downloaded from NCBI for comparison. KAAS web server (https://www.genome.jp/kaas-bin/kaas_main) for KEGG annotations was accessed on December 2019. The predicted genes were uploaded to the server, and then compared with the KEGG database of the server using the bi-directional best hit as the assignment method.

### Population history analysis

Pairwise sequentially Markovian coalescent (PSMC) (Li and Durbin 2011) was used to infer population size history of *S. knerii* from a diploid sequence. The whole genome diploid consensus sequence was generated as follows: ”BWA-MEM” algorithm from bwa v0.7.17 (Heng 2013) was used to map the 80x fastq data to the assembled genome of *S. knerii*. Then, SAMtools v0.1.19 (Li *et al.* 2009) was used to generate the diploid consensus with default settings (https://github.com/lh3/psmc), except that -d and -D were set as “-d 26 -D 160”. The default settings of PSMC were adopted. A generation time of 2.5 years for Siniperca and a substitution rate of 2.22 × 10-9 per site per year were set for the PSMC run (Liu *et al.* 2017).

### Data availability

The raw data are deposited in NCBI with SRA accessions numbers: SRR10231126 and SRR10231125. The BioSample is available with accession number SAMN12990280 at NCBI. The assembled genome also is available at NCBI and the accession number is WEHY00000000. The *S. chuatsi* full-length transcriptome data were downloaded from NCBI and the BioProject number is PRJNA552987. Supplemental material available at figshare: http://doi.org/10.6084/m9.figshare.11534487. All of the annotation files and supplemental files are available at https://github.com/LuLiangL/SK_anno.

## Results and Discussion

### Sequencing results and k-mer analysis

We obtained 373.3 million paired-end reads (56 Gbp data) after trimming low quality reads, in which 79.4% nucleotides had a quality score greater than 30. We used 28 Gbp of data for k-mer analysis. GenomeScope was used to generate a histogram of the depth distribution of the sequencing (k = 25) (Figure 2). The horizontal axis of the graph is the depth of k-mer coverage, and the vertical axis is the frequency of k-mer occurrence. A single k-mer coverage peak was observed at a depth of 42.2 x. Based on this, the genome size, repeat sequence ratio and heterozygosity were estimated. The genome size estimated with the k-mer method was 707.9 Mb, which is consistent the estimation by (Jianxun *et al.* 1991), 0.7 G 0.83 G. The repetitive content accounted for 12.6% of the genome. The heterozygosity was 0.155%. Coverage of the heterozygous site was 21.1x (Table S1). Detailed summary of repeated elements can be found in the output file of RepeatMasker (Table S2).

### Genome assembly and evaluation

Average length of the HMW-gDNA used for 10X Genomics DNA library construction was 44.6 kb. Average read number of each barcode was 416. Average insertion size was 383 bp. Average distance between SNPs was 777 bp. All statistic outputs from Supernova analyses can be found in the supplementary materials (Table S3). The *de novo* assembly was performed using 56 G of sequencing data. The final size of the assembled genome was 732.1 Mb, which is close to the estimated result by k-mer method or by using microspectrophotometer (Jianxun *et al.* 1991). A total of 7,989 scaffolds were obtained from the assembly, of which 81 scaffolds were larger than 1 Mb, accounting for 89.8% of the total genome size. The contig N50 and scaffold N50 of the genome were 60.4 kb and 12.64 Mb, respectively. The longest scaffold was 30.54 Mb. GC content of the genome was 40.4% (Table 1).

**Table 1:** *De novo* assembly from Supernova on the genome of *Siniperca knerii*

Metric	Value
Assembly Size	732.1 Mb
Number of scaffolds	7,989
Number of long scaffolds (>=1 Mb)	81
>=1 Mb scaffolds ratio	89.8%
Max length scaffold	30.54 Mb
Scaffold N50	12.64 Mb
Contig N50	60.4 kb
GC%	40.40%

Benchmarking Universal Single-copy Orthologs (BUSCO) v3.0.1 (BUSCO, RRID: SCR_015008) was used for genome integrity assessment. We used 4584 genes of actinopterigians for evaluation, of which 4424 genes (96.5%) were completely retrieved in the assembled genome, including 4320 (94.2%) single-copy genes and 104 (2.3%) multi-copy genes. In addition, 82 (1.8%) genes were fragmented and 78 (1.7%) genes were not found. BUSCO results show that most of the reference genes were assembled in the genome of *S. knerii*, demonstrating a high degree of genome integrity (Figire. S1).

### Gene prediction and annotation

The 28,440 predicted genes were distributed on 1,846 scaffolds, with an average of 15.4 genes per scaffold. The predicted coding genes were searched against existing databases, including InterProScan5 (Full results in Supplementary Table S4), Nr, KEGG, in which 91% of the genes obtained at least one hit (Table 2). Of the total 28,440 protein-coding genes, 25,899 (91.07%) genes have significant similarity to known coding genes in the NCBI database, 25,711 (80.05%) genes are annotated with InterProScan5, and 10,220 (35.9%) genes were retrieved by the KEGG pathway annotation.

**Table 2:** Comparing predicted genes of *Siniperca knerii* against the InterProScan5, Nr, and KEGG database

Metric	Value
Assembly Size	732.1 Mb
Number of scaffolds	7,989
Number of long scaffolds (>=1 Mb)	81

### Population history of S. knerii

**Figure 3:**
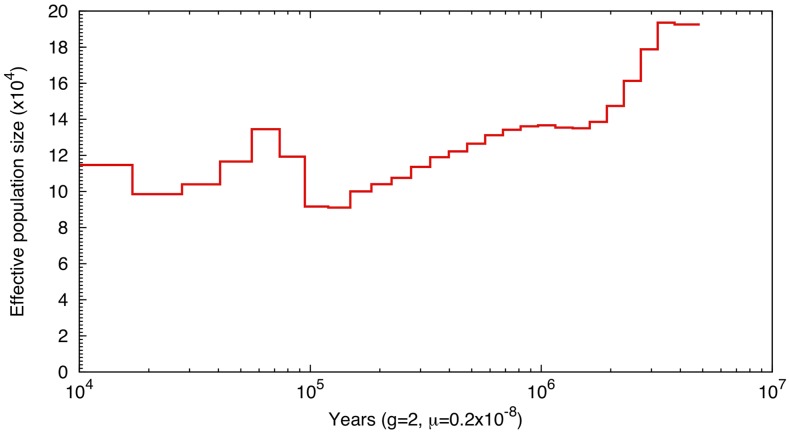
Fluctuation of population size of Siniperca knerii between 10 Ma to 10 ka.

We used PSMC analysis to estimate historic population dynamics of *S. knerii*. The effective population size of *S. knerii* between 10 ka and 10 Ma is shown in Figure 3. A trend of population declining is apparent from 10 Ma to 100 ka. Population size started increasing 100 ka, and peaked at 70 ka. The last interglacial period extended from 100 ka to 70 ka (Cui *et al.* 2011), which may cause population expansion of *S. knerii*. Then, there was a population decline after 70 ka, until before 20 ka, which is concordant with the Tali glacial period in eastern China (57 ka - 16 ka) (Wan *et al.* 2011).

## Conclusion

We leveraged 10x Genomics technology and paired-end Illumina sequencing to generate a draft genome assembly of the big-eye mandarin fish, *S. knerii*. We obtained high quality of genome assembly, with a scaffold N50 of 12.64 Mb and a genome completeness of 96.5%. The assembled *S. knerii* genome can be a fundamental resource for studying genetic variation of the species. As the first sequenced genome of the Sinipercidae, it can also be useful in comparative study of related fish families, such as the North American sunfish.
